# Prognostic Significance of the pN Classification Supplemented by Vascular Invasion for Esophageal Squamous Cell Carcinoma

**DOI:** 10.1371/journal.pone.0096129

**Published:** 2014-04-24

**Authors:** Chong-Mei Zhu, Yi-Hong Ling, Shao-Yan Xi, Rong-Zhen Luo, Jie-Wei Chen, Jing-Ping Yun, Dan Xie, Mu-Yan Cai

**Affiliations:** 1 Sun Yat-sen University Cancer Center, State Key Laboratory of Oncology in South China, Collaborative Innovation Center for Cancer Medicine, Guangzhou, China; 2 Department of Pathology, Sun Yat-sen University Cancer Center, Guangzhou, China; The University of Hong Kong, China

## Abstract

**Background:**

The biological behavior and clinical outcome of esophageal squamous cell carcinoma (ESCC) are difficult to predict.

**Methodology/Principal Findings:**

We investigate the prognostic impact of vascular invasion to establish a risk stratification model to predict recurrence and overall survival. We retrospectively evaluated the vascular invasion of 433 patients with ESCC treated with surgery between 2000 and 2007 at a single academic center. Those patients were assigned to a testing cohort and a validation cohort by random number generated in computer. The presence of vascular invasion was observed in 113 of 216 (52.3%) and 96 of 217 (44.2%) of ESCC in the training and validation cohorts, respectively. Further correlation analysis demonstrated that vascular invasion in ESCC was significantly correlated with more advanced pN classification and stage in both cohorts (*P*<0.05). Additionally, presence of vascular invasion in ESCC patients was associated closely with poor overall and recurrence-free survival as evidenced by univariate and multivariate analysis in both cohorts (*P*<0.05). In the subset of ESCC patients without lymph node metastasis, vascular invasion was evaluated as a prognostic predictor as well (*P*<0.05). More importantly, the combined prognostic model with pN classification supplemented by vascular invasion can significantly stratify the risk (low, intermediate and high) for overall survival and recurrence-free survival in both cohorts (*P*<0.05). The C-index to the combined model showed improved predictive ability when compared to the pN classification (0.785 *vs* 0.739 and 0.689 *vs* 0.650 for the training and validation cohorts, respectively; *P*<0.05).

**Conclusions/Significance:**

The examination of vascular invasion could be used as an additional effective instrument in identifying those ESCC patients at increased risk of tumor progression. The proposed new prognostic model with the pN classification supplemented by vascular invasion might improve the ability to discriminate ESCC patients’ outcome.

## Introduction

Esophageal cancer is the eighth most common cancer and the sixth leading cause of cancer death worldwide [1]. As the dominant type of esophageal cancer in China, esophageal squamous cell carcinoma (ESCC) distributes a general poor prognosis due to lack of a singular effective clinical method for early diagnosis. Despite improvements in its detection and treatment, the outcome in patients with ESCC remains poor, with an overall 5-year survival of 15–34% [2]–[3]. Most patients who undergo curative treatment for ESCC will eventually relapse and die as a result of this cancer. Given the poor outcome of ESCC and its high incidence, it is increasingly important to understand the progression of this cancer and to identify the most associated prognostic factors.

Appropriate risk stratified selection for adjuvant treatment trials is paramount, considering the high cost and toxic side effects of chemotherapeutic drugs. Different clinicopathological parameters such as tumor location, size, differentiation, infiltrative depth, lymph node involvement and distant metastasis, have been proposed as relevant factors to predict cancer-specific survival in patients with ESCC [4]. To improve the predictive accuracy of single prognosticator, the 7^th^ edition AJCC/IUAC TNM classification system comprising several clinicopathological features and predicting different outcomes have been constructed [5]. Although currently proposed TNM system shows considerable prognostic accuracy, there remains demand for increasing the accuracy of existing outcome predictive system.

Vascular invasion has been incorporated into TNM Classification of Malignant Tumors and College of American Pathologists Consensus Statement in pathological reports [6]–[8]. Assessment of vascular invasion with H&E staining enables identifying patients with high risk within the same TNM stage and therapeutic strategy can be tailored accordingly [9]. Since the report was investigated by Suqimachi et al. 1986 [10], numerous studies have been conducted on vascular invasion in ESCC. Most of these studies indicated that vascular invasion in ESCC was the relevant predictor of cancer-specific survival [4], [11]–[13]. Additionally, vascular invasion in superficial ESCC is also found to be a strong risk factor correlated with lymph node metastasis [14]. Therefore, it seems to be an attractive prognostic predictor. However, several clinicopathological features failed to improve the accuracy of existing multivariate prognostic models when they were analyzed for their added value [15]–[16].

The objective of the current study was to assess the prognostic value of vascular invasion in patients with ESCC, focusing on the predictive significance of the pN classification supplemented by vascular invasion in a large, Chinese, single center cohort of patients with ESCC.

## Materials and Methods

### Ethics Statement

The study was approved by the Institute Research Medical Ethics Committee of Sun Yat-sen University. No informed consent (written or verbal) was obtained for use of retrospective tissue samples from the patients within this study, most of whom were deceased, since this was not deemed necessary by the Ethics Committee, who waived the need for consent. All samples were anonymised.

### Patients and Cohorts

A total of 433 patients with ESCC, who underwent curative esophagectomy between October 2000 and May 2007, were randomly selected from the Department of Pathology of Sun Yat-sen University Cancer Center (Guangzhou, China). The selective criteria were: (1) having no adjuvant treatment before operation; (2) complete resection of the tumor; (3) incised margin was negative; (4) without distant metastasis; (5) follow-up data was detailed and complete. Those patients were assigned to a testing cohort and a validation cohort by random number generated in computer.

The training cohort was composed of 152 (70%) male and 64 (30%) female, with median age of 56.5 years. Average follow-up time was 41.3 months (median, 38.5 months; range, 1.0–115.0 months). In parallel, the validation cohort included 169 (78%) male and 48 (22%) female, with a median age of 57.0 years. Average duration of follow-up in this cohort was 42.3 months (median, 39.0 months; range, 2.0–106.0 months).

We collected clinicopathologic data including patient age, gender, tumor location, tumor size, differentiation, TNM stage, infiltrative depth, lymph node status, vascular invasion and recurrence. These data are detailed in Table 1. Tumor differentiation was determined based on the criteria proposed by WHO classification of Tumours of the Digestive System (2010 version). Tumor stage was defined according to the American Joint Committee on Cancer/International Union Against Cancer TNM (tumor-node-metastasis) classification system (2010 version). The patients were followed every 3 month for the first year and then every 6 months for the next 2 years and finally annually after surgery. The tumor recurrence (including local recurrence or metastasis) was detected by ultrasonography, CT or MRI. The time of detection of recurrence was still not known until the patient was dead of ESCC, and the time to death was used instead.

**Table 1:** Correlation of vascular invasion with patients’ clinicopathological features in primary esophageal squamous cell carcinomas.

Variable	Vascular invasion								
	Training cohort				Validation cohort				
	Cases	Absent	Present	*P* value*	Cases	Absent	Present	*P* value*	
Age (years)				0.941				0.021	
≤57.0†	118	56 (47.5%)	62 (52.5%)		112	54 (48.2%)	58 (51.8%)		
>57.0	98	47 (48.0%)	51 (52.0%)		105	67 (63.8%)	38 (36.2%)		
Gender				0.459				0.684	
Female	64	33 (51.6%)	31 (48.4%)		48	28 (58.3%)	20 (41.7%)		
Male	152	70 (46.1%)	82 (53.9%)		169	93 (55.0%)	76 (45.0%)		
Location				0.028				0.159	
Upper	10	3 (30.0%)	7 (70.0%)		18	11 (61.1%)	7 (38.9%)		
Middle	146	63 (43.2%)	83 (56.8%)		152	82 (53.9%)	70 (46.1%)		
Lower	60	37 (61.7%)	23 (38.3%)		47	28 (59.6%)	19 (40.4%)		
Tumor size (cm)				0.926				0.985	
≤4‡	137	65 (47.4%)	72 (52.6%)		140	78 (55.7%)	62 (44.3%)		
>4	79	38 (48.1%)	41 (51.9%)		77	43 (55.8%)	34 (44.2%)		
Differentiation				0.103				0.092	
Well	28	14 (50.0%)	14 (50.0%)		39	27 (69.2%)	12 (30.8%)		
Moderate	148	76 (51.4%)	72 (48.6%)		140	77 (55.0%)	63 (45.0%)		
Poor	40	13 (32.5%)	27 (67.5%)		38	17 (44.7%)	21 (55.3%)		
pT classification				0.033				0.268	
T1	13	9 (69.2%)	4 (30.8%)		7	6 (85.7%)	1 (14.3%)		
T2	55	32 (58.2%)	23 (41.8%)		49	27 (55.1%)	22 (44.9%)		
T3	148	62 (41.9%)	86 (58.1%)		161	88 (54.7%)	73 (45.3%)		
pN classification				0.002				0.002	
N0	120	61 (50.8%)	59 (49.2%)		113	76 (67.3%)	37 (32.7%)		
N1	48	30 (62.5%)	18 (37.5%)		63	29 (46.0%)	34 (54.0%)		
N2	37	10 (27.0%)	27 (73.0%)		34	15 (44.1%)	19 (55.9%)		
N3	11	2 (18.2%)	9 (81.8%)		7	1 (14.3%)	6 (85.7%)		
Stage				0.021				0.001	
I	13	8 (61.5%)	5 (38.5%)		12	11 (91.7%)	1 (8.3%)		
II	124	67 (54.0%)	57 (46.0%)		114	71 (62.3%)	43 (37.7%)		
III	79	28 (35.4%)	51 (64.6%)		91	39 (42.9%)	52 (57.1%)		

*Chi-square test;

† Median age;

‡ Median size.

### Pathological Evaluation

Patient records and original histopathologic slides were independently reviewed by 2 pathologists with special experience in gastrointestinal pathology (S.-Y. Xi and M.-Y. Cai) who were blinded to the pathological diagnoses and outcome data. Discrepancies were solved by simultaneous re-examination of the slides by both pathologists with a double-headed microscope. A mean of 4.2 (median 4, range 3–8) paraffin-embedded tissue blocks per tumor were available for evaluation, and all of these patients had at least 3 tissue blocks available.

The presence of vascular invasion was carefully evaluated on hematoxylin and eosin (H&E)-stained slides. Vascular invasion was defined as infiltration of vessel walls or the existence of tumor emboli [4]. The lymphatic channels were included in our study. Special care was taken to differentiate endothelial cells from retraction artifacts lined by fibroblasts.

### Statistical Analysis

The correlation between vascular invasion and the clinicopathologic features of the ESCC patients was evaluated by a χ2-test. For univariate analysis, survival curves were obtained with the Kaplan-Meier method, and the differences between groups in survival were tested by the log-rank test. Multivariate survival analyses were performed with the Cox proportional hazard regression model. The Harrell concordance index (C-index) was employed to assess model prognostic accuracy on multivariate analysis. A significant difference was deemed if the *P* value from a two-tailed test was less than 0.05. Statistical analysis was performed with SPSS statistical software package (SPSS Standard version 13.0; SPSS, Chicago, IL, USA) and R, version 3.0.1 (http://www.r-project.org/).

## Results

### The Patterns of Vascular Invasion

**Figure 1:**
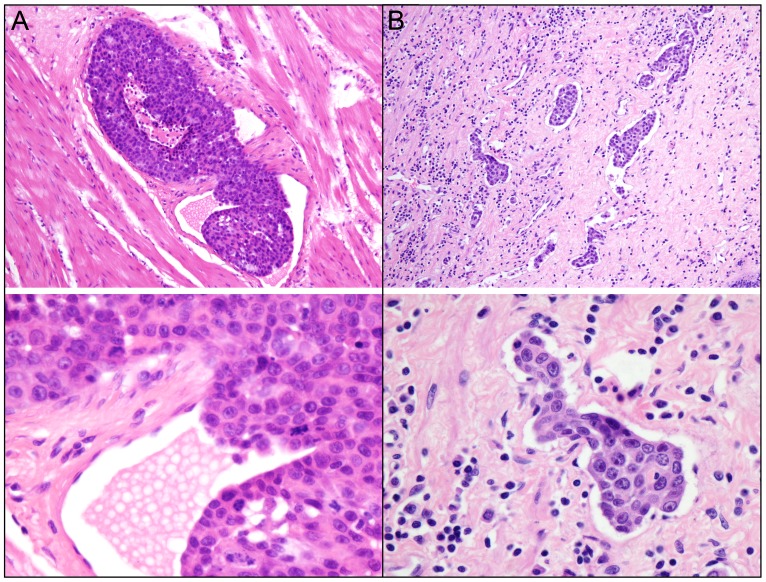
The patterns of vascular invasion in ESCC. A: Tumor cells were encroaching into the blood vessel wall (upper panel, ×100; lower panel, ×400). B: Tumor emboli were found in vascular spaces (upper panel, ×100; lower panel, ×400).

Vascular invasion in ESCC was identified as infiltration of vessel walls (Figure 1A) or the existence of tumor emboli in vascular spaces (Figure 1B). In the training cohort, presence of vascular invasion was observed in 113 of 216 (52.3%) of ESCCs. Further correlation analysis demonstrated that the presence of vascular invasion was significantly correlated with tumor location, infiltrative depth, pN classification and stage in ESCC (*P*<0.05, Table 1).

In the validation cohort, the presence of vascular invasion was found in 96 of 217 (44.2%) of ESCCs. Similar to the observations in the training cohort, presence of vascular invasion was correlated closely to certain clinicopathological features, including patient age, pN classification and stage (*P*<0.05, Table 1).

### Survival Analysis

**Figure 2:**
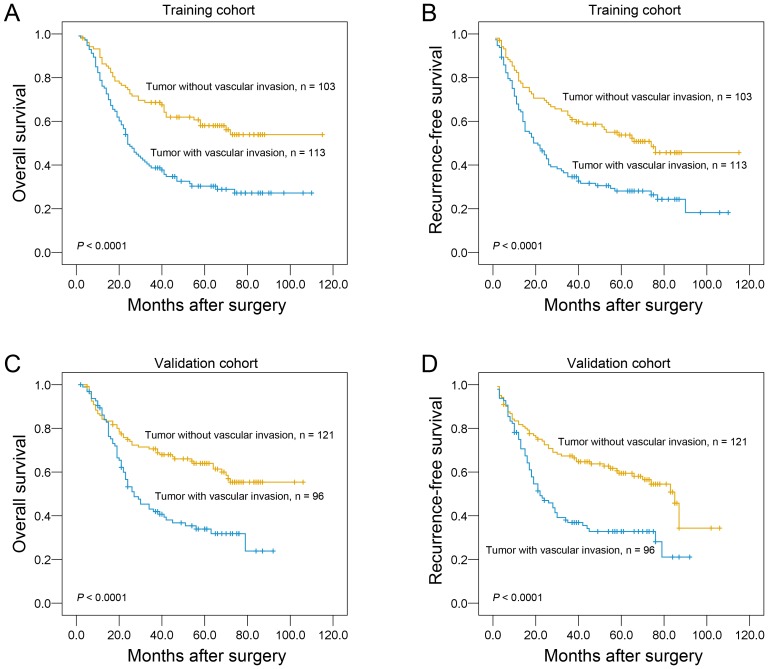
The association of vascular invasion with ESCC patients’ survival (log-rank test). Kaplan-Meier survival analysis of vascular invasion for overall survival (A) and recurrence-free survival (B) in the training cohort. Kaplan-Meier survival analysis of vascular invasion for overall survival (C) and recurrence-free survival (D) in the validation cohort.

Assessment of survival in the training cohort of ESCC patients revealed that some clinicopathological parameters indicated a significant impact of prognosis, such as infiltrative depth (*P*  = 0.005), pN classification (*P*<0.0001), tumor stage (*P*<0.0001) and vascular invasion (*P*<0.0001, Table 2). The result demonstrated that the patients with vascular invasion displayed a poor overall survival (Table 2; Figure 2A) and recurrence-free survival (Figure 2B) than the patients without vascular invasion (*P*<0.0001).

**Table 2:** Univariate analysis of vascular invasion and clinicopathologic variables in patients with primary esophageal squamous cell carcinoma (log-rank test).

Variable	Training cohort				Validation cohort			
	Cases	Mean survival (months)	Median survival (months)	*P* value	Cases	Mean survival (months)	Median survival (months)	*P* value
Age (years)				0.698				0.307
≤57.0*	118	61.6	42.0		112	57.4	45.0	
> 57.0	98	52.3	41.0		105	64.4	71.0	
Gender				0.122				0.043
Female	64	68.4	74.0		48	62.6	NR	
Male	152	55.4	33.0		169	58.0	51.0	
Location				0.191				0.354
Upper	10	71.5	NR		18	59.1	64.0	
Middle	146	50.0	31.0		152	59.1	46.0	
Lower	60	68.2	58.0		47	58.8	NR	
Tumor size (cm)				0.090				0.438
≤4†	137	64.5	53.0		140	54.9	64.0	
>4	79	45.4	29.0		77	58.3	54.0	
Differentiation				0.316				0.033
Well	28	49.3	47.0		39	67.7	NR	
Moderate	148	63.4	47.0		140	63.2	71.0	
Poor	40	44.8	25.0		38	40.0	27.0	
pT classification				0.005				0.093
T1	13	52.9	NR		7	67.1	NR	
T2	55	72.9	74.0		49	65.0	70.0	
T3	148	53.4	27.0		161	58.3	51.0	
pN classification				0.000				0.000
N0	120	80.2	NR		113	73.2	NR	
N1	48	38.2	31.0		63	52.3	51.0	
N2	37	22.1	15.0		34	32.7	24.0	
N3	11	12.5	9.0		7	29.4	23.0	
Stage				0.000				0.000
I	13	52.6	NR		12	85.1	NR	
II	124	79.1	NR		114	68.8	NR	
III	79	24.3	16.0		91	43.5	30.0	
Vascular invasion				0.000				0.000
Absent	103	74.8	NR		121	71.9	NR	
Present	113	46.2	24.0		96	44.0	27.0	

*Median age;

† Median size; NR indicates not reached.

Results in the validation cohort were similar to those in the training cohort. Patients with presence of vascular invasion also showed a significant trend toward worse overall survival (Table 2; Figure 2C) and recurrence-free survival (Figure 2D) compared to the patients with absence of vascular invasion (*P*<0.0001). Of the other prognostic factors, univariate analysis showed that gender (*P*<0.05), differentiation (*P*<0.05), lymph node status (*P*<0.0001) and tumor stage (*P*<0.0001) adversely affected patient disease-specific survival (Table 2).

**Figure 3:**
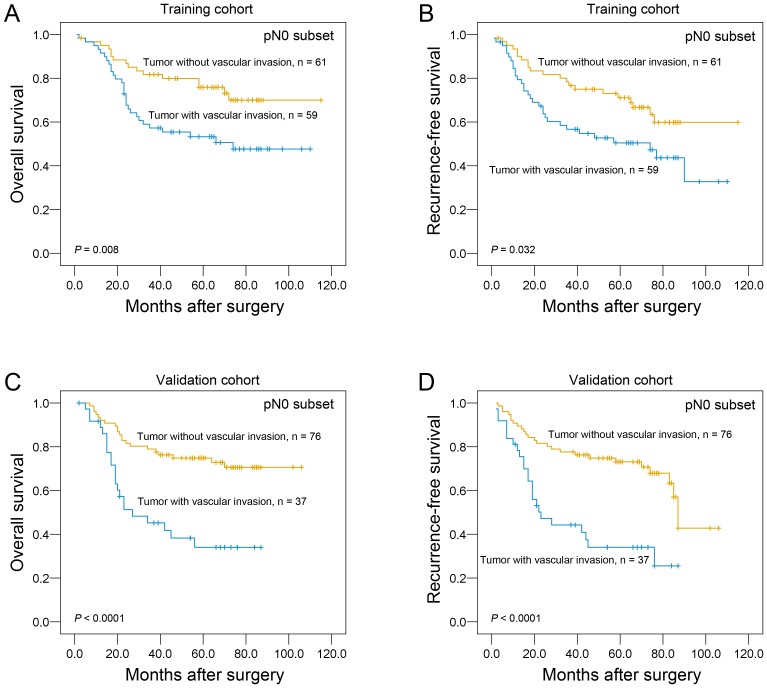
Univariate survival analysis with regard to vascular invasion in the subset of ESCC patients with pN0 classification. The presence of vascular invasion was identified as a prognostic predictor of overall survival and recurrence-free survival in ESCC patients without lymph node metastasis in training cohort (A and B) and validation cohort (C and D).

Further survival analysis was performed with regard to vascular invasion in the subset of ESCC patients without lymph node metastasis. Our results demonstrated that the presence of vascular invasion was identified as a prognostic predictor of overall survival and recurrence-free survival in ESCC patients without lymph node metastasis in both cohorts (*P<*0.05, Figure 3).

### Multivariate Cox Regression Analysis on the Two Cohorts

Since variables examined to have prognostic influence by univariate analysis may covariate, the presence of vascular invasion as well as other clinicopathologic features (including tumor size, differentiation, infiltrative depth, pN status and stage) were tested in multivariate analysis (Table 3). In the training cohort, the presence of vascular invasion was found to be a significantly independent prognostic factor for poor overall survival (hazard ratio, 1.622; 95% CI, 1.096–2.401; *P*  = 0.016; Table 3). Similar results were also observed in our validation cohort (hazard ratio, 1.655; 95% CI, 1.100–2.489; *P*  = 0.016; Table 3). Of the other parameters, pN classification was evaluated as an independent prognostic factor for patient survival in both cohorts.

**Table 3:** Cox multivariate analyses of prognostic factors for overall survival.

Variables	Hazards ratio	95% CI	*P* value
**Training cohort**			
Tumor size, cm (≤4* *v* >4)	1.134	0.783–1.643	0.506
Differentiation (well *v* moderate *v* poor)	1.103	0.816–1.490	0.525
pT classification (T1 *v* T2 *v* T3)	1.113	0.726–1.706	0.622
pN classification (N0 *v* N1 *v* N2 *v* N3)	1.759	1.260–2.455	0.001
Stage (I *v* II *v* III)	1.505	0.771–2.937	0.231
Vascular invasion (absent *v* present)	1.622	1.096–2.401	0.016
**Validation cohort**			
Tumor size, cm (≤4 *v* >4)	1.104	0.747–1.633	0.619
Differentiation (well *v* moderate *v* poor)	1.181	0.853–1.634	0.317
pT classification (T1 *v* T2 *v* T3)	1.421	0.900–2.241	0.131
pN classification (N0 *v* N1 *v* N2 *v* N3)	1.512	1.087–2.103	0.014
Stage (I *v* II *v* III)	0.924	0.513–1.663	0.791
Vascular invasion (absent *v* present)	1.655	1.100–2.489	0.016

*Median size; CI, confidence interval.

### New Prognostic Model with pN Classification Supplemented by Vascular Invasion

**Figure 4:**
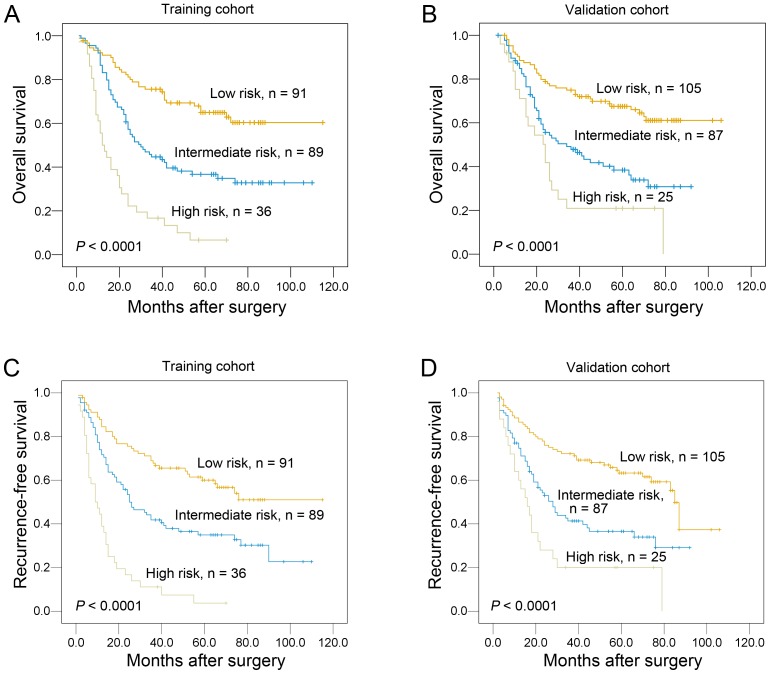
Comparison of overall survival according to a new combined prognostic model. The new combined model (including pN classification and vascular invasion) could significantly stratify the risk (low, intermediate and high) for overall survival of ESCC patients in training cohort (A) and validation cohort (B). The proposed model could significantly discriminate the risk (low, intermediate and high) for recurrence-free survival of ESCC patients in training cohort (C) and validation cohort (D).

According to the results of our multivariate analyses, we proposed a new clinicopathologic prognostic model with 2 prognostic factors, i.e., pN classification and vascular invasion. We designated a high-risk group as the presence of the advanced pN classification (2 or 3) and vascular invasion, an intermediate-risk group as the presence of one factor [the presence of the advanced pN classification (2 or 3) or vascular invasion], and a low-risk group as the presence of none [the low pN classification (i.e., pN0 or pN1) and absence of vascular invasion]. Our results revealed that the proposed model could significantly stratify the risk (low, intermediate and high) for overall survival (Figure 4A and 4B, *P*<0.0001) and recurrence-free survival (Figure 4C and 4D, *P*<0.0001) in both cohorts.

In training cohort, application of C-index to the proposed new prognostic model showed improved predictive ability when compared with the single pN classification model (c indexes of 0.785 *vs* 0.739, respectively). Similar finding was observed in the validation cohort, the C-index of the pN classification supplemented by vascular invasion was 0.689 compared to 0.650 in our validation study of the pN classification model.

## Discussion

It has been suggested that the 5-year overall survival of ESCC is 15–34% [2]–[3] and suitable and individual management of ESCC is needed to improve the outcome for patients. TNM stage is traditionally considered the single most important prognostic factor of ESCC. Other features have been found to be prognostic assessment of patients with ESCC. In this regard, the presence of tumor size, differentiation, location, performance status of patient, tumor infiltration depth, lymph node status and distant metastasis have a major role, and are extensively utilized in clinical setting [4], [10]–[11], [14], [17]–[23].

In the current study, we assessed a retrospective collection of data on patients with ESCC to determine the prognostic accuracy of the pN classification compared to this classification supplemented by vascular invasion. Our results demonstrated that in testing and validation cohorts, the presence of vascular invasion was frequently observed in ESCC as evaluated on H&E-stained slides. Further correlation analyses in testing cohort revealed that the presence of vascular invasion in ESCCs was significantly associated with tumor location, infiltrative depth, pN status and stage. Similar result was confirmed in our validation cohort. In addition, multivariate analyses in both cohorts evaluated that the presence of vascular invasion was a prognostic factor independent of certain well-established clinical factors, including tumor size, differentiation, pT status, pN status and clinical stage. The C-index analysis showed that the proposed new prognostic model (combined pN classification and vascular invasion) could improve the predictive ability when compared to pN classification.

Our findings of vascular invasion status and its correlation with ESCC patients’ outcome are consistent with the results of other groups. In 1986, Sugimachi et al found a significant association between the presence of vascular invasion and 5-year survival as evidenced by univariate analysis [10]. A similar result was reported by Ide et al. [11], in which multivariate analysis of ESCC patients revealed that vascular invasion was a significant prognostic predictor of the overall survival. In a more recent study, Kitagawa et al. investigated the prognostic value of epidermal growth factor receptor (EGFR) gene amplification in patients with ESCC and found that vascular invasion was proved to retain independent prognostic value [12]. Importantly, vascular invasion in superficial ESCC is also found to be a strong independent predictor of lymph node metastasis with an odds ratio of 12.01 [14]. Notably other published reports show no significantly prognostic value for vascular invasion to predict the outcome in patients with ESCC [15]–[16]. Taken together, differences in clinicopathologic characteristics among cohorts, geographic backgrounds, methodology in vascular invasion detection, patient heterogeneity, lack of independent validation of the results, small sample size and different definitions of end points (disease-free, cancer specific or overall survival) might contribute to the controversial results.

Generally, our findings support the idea that the pN classification supplemented by vascular invasion might improve the ability to discriminate ESCC patients’ outcome, especially in the patients without lymph node metastasis. As it is well known that the pTNM stage and tumor differentiation are the best-established risk factors for important aspects affecting the prognosis of patients with ESCC. These two variables, based on specific clinicopathologic features and extent of disease, may have reached their limits in providing critical information influencing patient prognosis and treatment strategies. Furthermore, outcome of patients with same stage following surgery is substantially different and such large discrepancy has not been well understood [24]–[25]. Thus, there is a need for new objective strategies that can effectively distinguish between patients with favorable and unfavorable outcome. In the present study, our data support the concept that vascular invasion, as detected by H&E staining, can identify ESCC patients with or without aggressive clinical course and/or poor outcome. Thus, evaluation of vascular invasion may become a factor for predicting prognosis and rendering a more tailored treatment strategy in patients with ESCC. Based on our results, we proposed a new prognostic model with pN classification supplemented by vascular invasion. This combined model can reflect the aggressive phenotype of ESCC. There are also strong efforts to integrate biomarkers into established clinicopathologic models to further improve their predictive ability [26]. Thus, this combined model may be a useful prognostic index for ESCC.

Although the present study was retrospective, it was strengthened by the fact that all histopathologic slides were reviewed by two gastrointestinal pathologists. Moreover, all classical clinicopathologic features were re-evaluated and compared to vascular invasion, which stood out as the most relevant predictor of ESCC aggressiveness. At last, we proposed a new prognostic model combining pN classification with vascular invasion that could be easily determined by the pathologists and used to accurately predict the biological behavior of ESCC. To our knowledge, this is the first report to investigate the prognostic ability of the pN classification supplemented by vascular invasion; however, further external validation of this important model is needed using pooled multicenter data.

In the present study, we observed that presence of vascular invasion was a strong and independent predictor of adverse survival, as evidenced by Kaplan-Meier curves and multivariate Cox proportional hazard regression analysis. The proposed new prognostic model with the pN classification supplemented by vascular invasion might improve the ability to discriminate ESCC patients’ outcome. Thus, the examination of vascular invasion could be used as an additional effective instrument in identifying those ESCC patients at increased risk of tumor progression. This instrument might also help the clinician to choose a suitable therapy for the individual patient, for example, favoring a more aggressive treatment in patients with vascular invasion.
